# Comprehensive analysis of telomerase inhibition by gallotannin

**DOI:** 10.18632/oncotarget.24642

**Published:** 2018-04-10

**Authors:** Nikita Savelyev, Polina Baykuzina, Svetlana Dokudovskaya, Olga Lavrik, Maria Rubtsova, Olga Dontsova

**Affiliations:** ^1^ Lomonosov Moscow State University, Department of Chemistry, and A.N. Belozersky Institute of Physico-Chemical Biology, Moscow, 119992, Russia; ^2^ Lomonosov Moscow State University, Faculty of Bioengineering and Bioinformatics, Moscow, 119992, Russia; ^3^ UMR 8126, CNRS, University Paris-Sud, Université Paris Saclay, Institut de Cancérologie Gustave-Roussy, 94805, Villejuif, France; ^4^ Institute of Chemical Biology and Fundamental Medicine, Siberian Branch, Russian Academy of Sciences, Novosibirsk, 630090, Russia; ^5^ Novosibirsk State University, Novosibirsk, 630090, Russia; ^6^ Skolkovo Institute of Science and Technology, Skolkovo, Moscow region, 143025, Russia

**Keywords:** telomerase, telomere, gallotannin, inhibition, poly(ADP-ribosy)lation

## Abstract

Gallotannin (or tannic acid) is a naturally occurring compound that inhibits cell growth and activity of different DNA-polymerases, including telomerase. The purpose of the present study was to gain insight into the mechanism of telomerase inhibition by gallotannin. We determined that gallotannin inhibits telomerase *in vitro* with an half maximal inhibitory concentrations value of 130 nM, but it does not affect telomerase complex assembly and component levels *in vivo*. The inhibitory activity of gallotannin against telomerase provides an additional explanation for the anti-cancer activities of this compound.

## INTRODUCTION

Telomeres protect the ends of linear eukaryotic chromosomes from loss of genetic information during genome replication [1]. Telomerase is the enzyme that maintains the length of telomeres in germ, stem, cancer and somatic cells with increased proliferative potential [1, 2]. Telomerase contains two core components: telomerase reverse transcriptase (TERT) and telomerase RNA (TER). TERT uses TER as a template for the processive synthesis of telomere repeats at protruding 3’-ends of linear eukaryotic chromosomes [3, 4, 5]. Telomerase is active in the majority of cancer cells and is considered a universal target for anticancer therapy [1].

Different classes of chemical compounds have been described as telomerase inhibitors [6]. For example, oligonucleotides that mimic telomere sequences are recognized by telomerase as substrates [7]. This prevents the association of telomerase with telomeres and results in stable telomere shortening. The extended telomere length in cancer cells and a number of unspecific effects limits the application of this type of inhibitor [8, 9]. Another class of telomerase inhibitors is naturally occurring compounds [10, 11]. The most common sources of different biologically active components are medicinal herbs and food products. Many products, including tea, wine, coffee, chocolate, olives, fruits, vegetables, and nuts [12], contain condensed tannins, which possess antioxidant activity [13, 14], induce apoptosis in cancer cells [15, 16] and inhibit telomerase activity [17].

Gallotannin, also known as tannic acid, is found in several plants used for medical purposes such as *Caesalpinia spinosa* [18], *Rhus chinensis* [19] and *Quercus infectoria* [20]. Gallotannin is a direct inhibitor of poly(ADP-ribose)glycohydrolase (PARG) [21, 22], but it also suppresses DNA polymerases [23] and the proteasome [24]. In addition, gallotannin can also induce senescence [25] and the expression of inflammatory genes and cytokines [26, 27, 28]. Gallotannin treatment causes an anti-metastatic effect, as was revealed in a murine model [29]. Finally, it was shown recently that gallotannin inhibits telomerase activity *in vitro* [17], *in vivo* and in a murine model [30], but the molecular mechanisms of this inhibition are unknown. Here, we show that treatment of human cell lines with gallotannin inhibits telomerase activity both *in vitro* and *in vivo* but does not have an impact on the expression level of telomerase components and the assembly of the enzyme.

## RESULTS

### Gallotannin inhibits telomerase *in vivo* and *in vitro*

**Figure 1:**
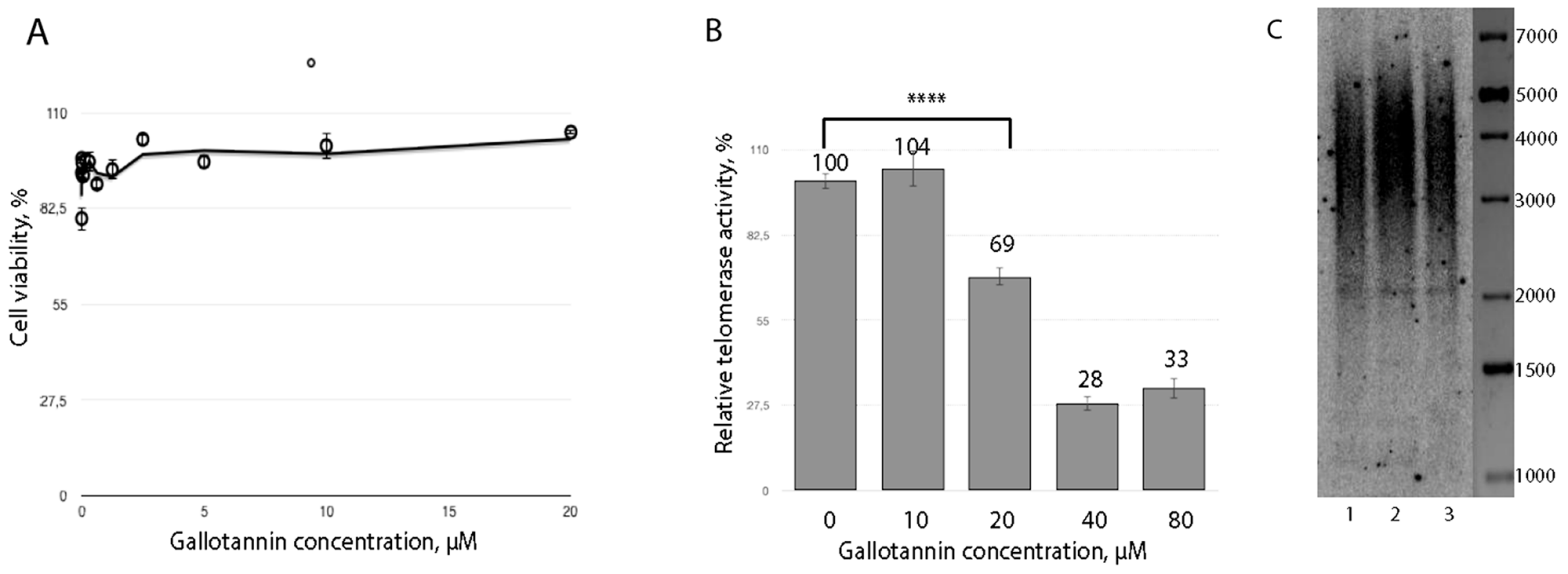
Gallotannin inhibits telomerase *in vivo* **(A)** Cytotoxicity of gallotannin in HEK293T cells treated with various concentrations of the compound (1,25 nM, 2,5 nM, 5 nM, 10 nM, 20 nM, 40 nM, 80 nM, 160 nM, 320 nM, 650 nM, 1,25 μM, 2,5 μM, 5 μM, 10 μM, 20 μM). **(B)** Telomerase activity measured in extracts from cells treated with various concentrations of gallotannin. **(C)** Telomere restriction fragment analysis of HEK293T cells treated with 2,5 μM (1) and 0,4 μM (2) gallotannin and untreated (3).

Treatment of HEK293T cells with gallotannin for 24 hours did not have an effect on cell viability for all concentrations tested (Figure 1A), in agreement with previously published data [31, 32], while treatment for 36 and 48 hours resulted in increased detachment and cell death. Therefore, we analyzed the influence of gallotannin on telomerase activity after 24 h of treatment. We observed significant inhibition of telomerase in cells treated with 40 μM and more of gallotannin (Figure 1B). We further cultivated HEK293T cells for 50 population doublings (PD) with various concentrations of gallotannin and analyzed telomere length by the telomere restriction fragments (TRF) technique. The cells treated with 2,5 μM and 400 nM of gallotannin for 50 PD did not show any differences in telomere length (Figure 1C).

**Figure 2:**
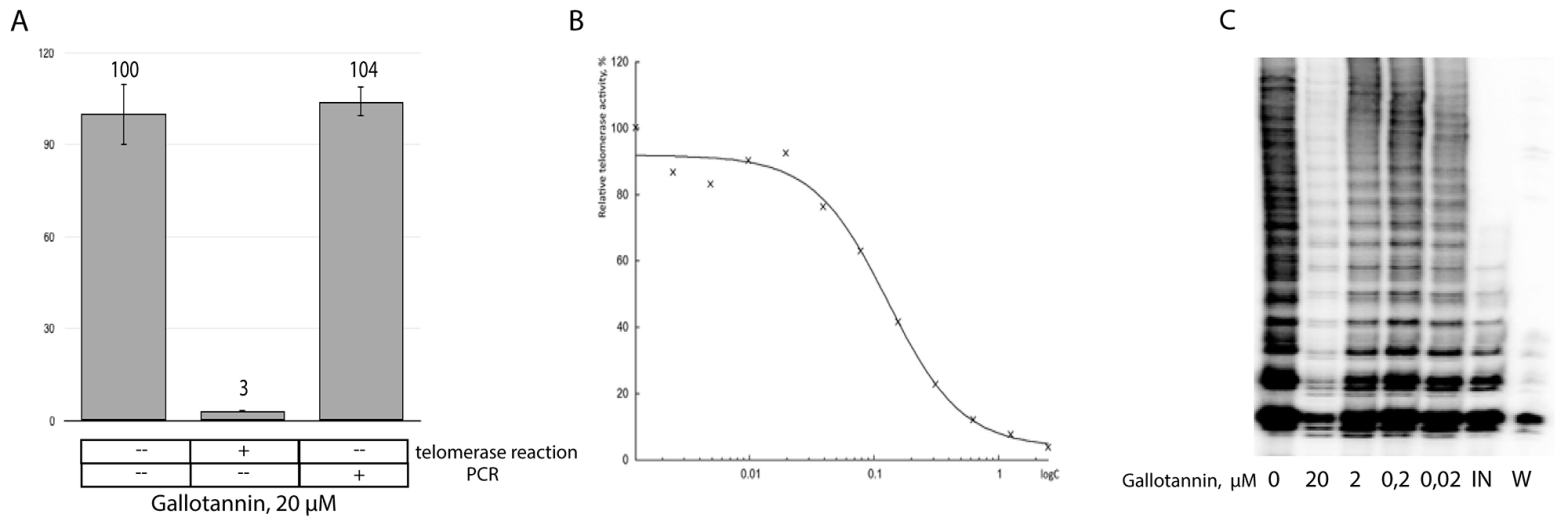
Gallotannin inhibits telomerase *in vitro* **(A)** RQ-TRAP analysis of partially purified telomerase. Gallotannin was added in the telomerase reaction or in the PCR step of the TRAP-assay. **(B)** Telomerase activity measured by RQ-TRAP in the presence of various concentrations of gallotannin. **(C)** Inhibitory effects of various concentrations of gallotannin (0, 02, 0,2, 2, 20 μM) on the processivity of telomerase. Telomerase activity was measured by TRAP assay in the presence of α[P^32^]-dATP followed by PAGE separation of DNA fragments. Abbreviations: IN, telomerase was inactivated by heating; W, To exclude contamination. TRAP assay was performed in the presence of water instead of cell lysates.

We further decided to check whether gallotannin inhibits telomerase directly because inhibition effect of gallotannin on many cellular polymerases was shown previously [23]. To test telomerase activity *in vitro* we used classical Quantitative Telomerase Repeat Amplification Protocol (RQ-TRAP), based on qPCR amplification of a DNA substrate elongated by telomerase, partially purified from a cell-free extract of HEK293E cells transfected with the plasmids overexpressing both hTR and hTERT. The telomerase elongation reaction was carried out in the presence of 20 μM of gallotannin, a concentration that does not inhibit *Taq*-polymerase when used in the RQ-TRAP assay (Figure 2A). The half maximal inhibitory concentration (IC_50_) of gallotannin was calculated to be 128,9 ± 18,5 nM (Figure 2B), demonstrating that this compound is an effective telomerase inhibitor.

Telomerase inhibitors may affect both polymerase activity and processivity. RQ-TRAP allows for the quantification of telomerase activity but does not show whether its processivity is blocked. To check telomerase processivity, we performed a radioactive TRAP assay (Figure 2C). While no influence on telomerase processivity was detected, the activity of telomerase was inhibited with the lowest concentration of gallotannin (0,2 μM).

### Gallotannin treatment has no impact on human telomerase component’s expression level and does not affect telomerase complex assembly

The effect of gallotannin on telomerase activity may be due to the direct interaction of the compound with the enzyme, the decreased expression level of complex components, or defects in telomerase assembly.

**Figure 3:**
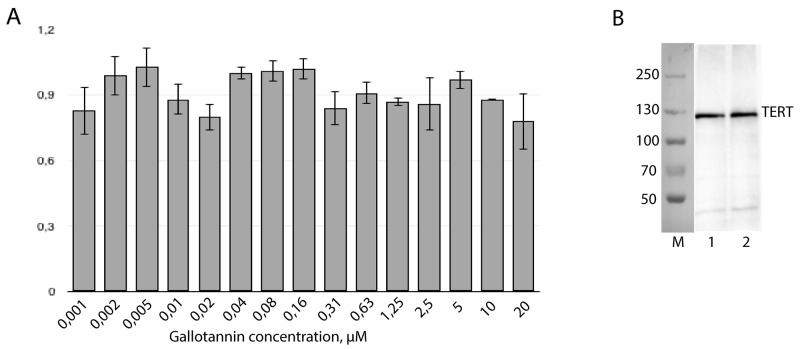
Gallotannin treatment has no impact on human telomerase components expression level **(A)** Amount of hTR was measured by RT-qPCR. Level of hTR in cells treated with gallotannin was normalized to the level of hTR in untreated cells after normalization to the *GAPDH* mRNA level. **(B)** Western-blotting analysis of hTERT in HEK293T cells (1) and HEK293T cells treated with 20 μM of gallotannin (2).

To check the influence of gallotannin on the expression level of telomerase components, RT-qPCR and Western blotting analyses of cellular extracts were performed. The level of hTR was unchanged in cells treated with gallotannin in the concentration range from 1,2 nM to 20 μM, as revealed by RT-qPCR (Figure 3A). The hTERT expression level was also unaffected (Figure 3B). Gallotannin is known as an inhibitor of PARG that cleaves branches of poly(ADP-ribose) synthesized by poly(ADP)-ribose polymerases (PARP) [22]. The treatment of cells with gallotannin should increase the level of poly(ADP-ribosyl)ation (PARylation) proteins as a result of PARG inhibition. PARylation should increase the molecular weight of the modified proteins, which may be detected by Western blotting as a shift of the initial protein band. The unchanged molecular weight of hTERT after gallotannin treatment (Figure 3B) allows us to infer that the activity hTERT is not regulated via PARylation at the conditions used in the experiments.

**Figure 4:**
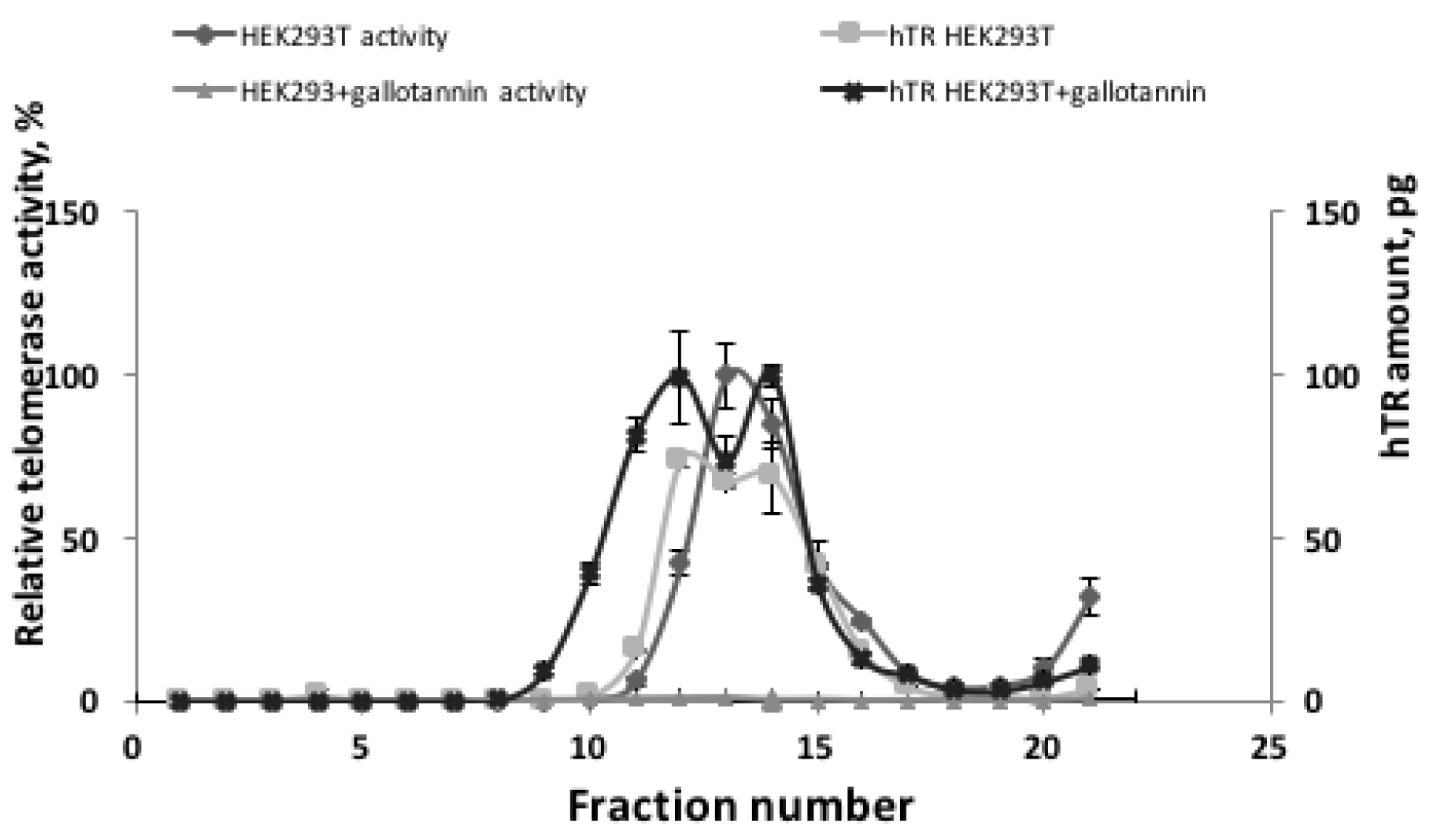
Gallotannin does not affect telomerase complex assembly Cellular lysates from HEK293T cells and HEK293T cells treated with 20 μM of gallotannin were fractionated by ultracentrifugation in sucrose concentration gradient. Obtained fractions were analyzed for telomerase activity and hTR level.

To test whether gallotannin treatment influences telomerase assembly, extracts obtained from cells treated with 20 μM of gallotannin were subjected to sucrose gradient centrifugation to separate assembled telomerase complexes from free hTR (Figure 4). In the control HEK293T cells as well as in cells treated with gallotannin, we observed that the peak of hTR correlates with the peak of telomerase activity. These data suggest that gallotannin does not affect telomerase assembly.

## DISCUSSION

Tannic acid and its derivatives inhibit many intracellular components and affect cell proliferation. Telomerase inhibition by tannic acid was demonstrated previously [17], and we confirmed these findings. The blocking of telomerase activity was proposed as the major mechanism of tumor growth inhibition by dietary polyphenols in a murine model [30]. The inhibition of telomerase may occur due to a decreased amount of telomerase components in cells. However, our data demonstrate that gallotannin does not change the amount of hTR and hTERT in HEK293T cells. Interestingly, a decreased expression level of known hTERT inhibitor NF-kB during gallotannin treatment was observed in A549 cells lines derived from human lung carcinoma [26].

Gallotannin is known as an inhibitor of PARG that cleaves branches of poly(ADP-ribose) synthesized by PARP enzymes [22]. Poly(ADP-ribosy)lation is the posttranslational modification that provides proteins with covalently attached negatively-charged and branched polymers of poly(ADP-ribose). This modification may influence protein-nucleic acid interactions [33]. The PARylation of hTERT [34] and telomerase-associated regulatory protein TEP1 [35] was shown previously in HeLa cells. Our data speak in favor of the view that hTERT modification was not observed in HEK293T cells under gallotannin treatment.

The efficiency of telomerase complex assembly is important for telomerase activity regulation [36] and might be a target for the development of telomerase inhibitors [37]. Telomerase assembly may be affected either by direct interaction of the inhibitor with telomerase components or due to the interaction of the inhibitor with additional cellular proteins that regulate telomerase complex formation [38]. Insufficient telomerase complex assembly will decrease the level of active telomerase in cells despite the unaffected amount of its components. For example, mutations of dyskerin, involved in telomerase complex assembly, lead to decreased levels of telomerase complex and telomerase activity [39]. The influence of gallotannin on telomerase complex assembly was not detected in our experiments, which allowed us to consider that gallotannin may directly inhibit telomerase polymerization activity.

The data on DNA polymerase activity inhibition by the derivatives of tannic acid were obtained previously [23]. Gallotannin inhibits the activity of DNA polymerase α, β and κ with IC_50_ values of 13, 130 and 30 nM, respectively. A docking simulation of gallotannin on the DNA polymerase β active site revealed its interaction with the catalytic pocket/binding site for the incoming dNTPs. This interaction affects the structure of the catalytic core of DNA polymerase and its consequent catalytic activity [23]. We observed the inhibition of telomerase by gallotannin, with an IC_50_ value of 128,9 ± 18,5 nM, that is comparable with the IC_50_ value determined for gallotannin competitive inhibition of DNA polymerase β. However, gallotannin inhibits the activity of telomerase *in vivo* at concentrations of 20-40 μM. The difference between inhibitory concentrations of gallotannin in *in vitro* and *in vivo* systems may be explained by the partial hydrolysis of this compound in the cell, as gallotannin is known as a hydrolysable tannin [23, 40].

The fact that gallotannin inhibits the activity of a wide range of key cellular enzymes, along with our data on the inhibition of telomerase, makes it a prospective anticancer drug. Telomerase inhibition by gallotannin will induce telomere shortening that is necessary for a synergetic inhibitory effect on cancer cell growth. Our findings suggest that gallotannin may be a prospective candidate drug to be used for cancer therapy either as an individual component or in combination with other medical compounds.

## MATERIALS AND METHODS

### Cell culture

The human embryonic kidney cell line HEK293T was purchased from the ATCC and maintained in Dulbecco’s modified Eagle’s medium (DMEM), supplemented with 10% heat-inactivated fetal bovine serum (FBS), 100 Units/mL penicillin, and 100 mg/mL streptomycin at 37°C in a humidified incubator with 5% CO_2_.

### Materials

The following agents and reagents were obtained: gallotannin from Sigma (#16201), CellTiter 96 Aqueous One Solution Cell Proliferation Assay kit from Promega (G3580), Pure Link RNA Mini kit from Ambion (#12183025), SYBR green I from Thermo Fischer Scientific (S7567), Maxima First strand cDNA synthesis kit from Thermo Fisher Scientific (K1642), anti-TERT antibodies from Abcam (ab183105), and anti-rabbit HRP conjugated antibodies from Thermo Fisher Scientific (G21234). Partially purified telomerase was obtained as described in [36].

### Cell proliferation assay

For the MTS assay, the CellTiter 96 Aqueous One Solution Cell Proliferation Assay kit was used according to the manufacturer’s instructions. HEK293T cells were plated in 96-well plates at a density of 1×10^4^ cells/well. After overnight incubation, the cells were treated with various concentrations of gallotannin for 24 hours. Untreated cells were used as a control. Absorbance at 490 nm in each well was recorded using a Victor plate reader (PerkinElmer) and normalized to the PBS-treated cells as 100% of viability. Each experiment was performed in triplicate.

### Telomere restricted fragment (TRF) analysis

Genomic DNA from 3×10^6^ cells was extracted with lysis buffer (200 mM Tris-HCl, pH 8.0, 100 mM EDTA, 1% SDS, 150 mg Proteinase K) at 55°C overnight. Then, 30 μg of genomic DNA was digested with both 20 U *Rsa*I and 20 U *Hinf* I for 16 hours at 37°C. The products of digestion were resolved at 0.7% agarose gel and in-gel hybridized with a telomere-specific probe as previously described [41].

### Telomerase assembly test

The telomerase assembly test was performed as described in [36] with minor modifications. 1×10^7^ cells were grown in the presence or absence of gallotannin for 24 hours, harvested, washed with ice-cold PBS, resuspended in the ice-cold CHAPS lysis buffer (HEPES pH 7.5 10 mM, EGTA 1 mM, MgCl_2_ 1 mM, glycerol 10%, CHAPS 0,5%, β-MeOH 5 mM, PMSF 0,1 mM) up to a final concentration of 1000 cells/μl and lysed for 30 min in ice. Cellular debris was removed by centrifugation at 16,000 g for 20 min, and the supernatant was loaded in the sucrose gradient obtained by two freeze-thaw cycles (−20°C - RT) of 20% sucrose in 1x TRAP buffer (TRIS pH 8.3 20 mM, MgCl_2_ 1,5 mM, KCl 63 mM, EGTA 1 mM, BSA 0,1 mg/ml, Tween 20 0,005%) either with or without 20 μM gallotannin. Centrifugation was performed in a SW-41 Ti rotor (Beckman) at 4°C for 20 h at 30000 rpm, and fractions were collected from the top. One-fifth volume of each fraction was set aside for the RQ-TRAP assay, and the rest was used for hTR quantification.

### TRAP and RQ-TRAP assays

Classical TRAP assay was performed as previously described [42]. Telomerase extracts from 2000 cells in CHAPS lysis buffer were added to TRAP buffer containing 100 ng of TS primer (5′-AATCCGTCGAGCAGAGTT-3′) and 2μCi α-^32^P-dATP. After 30 min of incubation the mixture containing 100 ng of ACX primer (5′-CCCTTACCCTTACCCTTACCCTTA-3′), *Taq*-polymerase was added, and amplification of DNA products was performed on a Mastercycler(Eppendorf). Amplification products were purified by phenol/chloroform extraction, dissolved in formamide dye and separated on 10% sequence PAGE. The gel was exposed to a Phosphor-imager screen and scanned using a Typhoon FLA 9500 (GE Healthcare). The RQ-TRAP assay was performed as a classical TRAP but with the addition of SYBR green I. Amplification and detection were performed on a CFX96 Real-Time System (Bio-Rad) [43].

### hTR quantification by RT-qPCR

RNA from each fraction after ultracentrifugation or total RNA from the cells grown in the presence of gallotannin was extracted using the Ambion Pure Link kit according to the manufacturer’s instructions. Reverse transcription was performed with the Maxima First strand cDNA synthesis kit, and the PCR reaction was made with hTR-F1 (5′-GTGGTGGCCATTTTTTGTCTAAC-3′) and hTR-R1 (5′-TGCTCTAGAATGAACGGTGGAA-3′) primers in a CFX-96 Real-Time System (Bio-Rad) for 30 amplification cycles. For telomerase assembly analysis, the hTR RNA T7-transcript was used as a standard for quantification [36]. The amount of hTR in cells treated with gallotannin was normalized to the hTR in untreated HEK293T cells.

All statistical calculations were performed with GraphPad Prism 6 software using column statistics or a one-way ANOVA Dunett test.

### IC_50_ calculation

For IC_50_ calculations, serial 2-fold dilutions of gallotannin in the RQ-TRAP assay were used. The values were normalized to those in HEK293T cells. IC_50_ was calculated with the online IC_50_ calculation tool (http://www.ic50.tk/).

### Western blotting

Cells were grown either in the absence or in the presence of gallotannin, lysed for 30 min in ice-cold RIPA buffer with 0.2 mM PMSF and centrifuged at 4°C for 15 min. The supernatant was collected, protein concentration was measured by the Bradford assay, and proteins were resolved in 8% PAGE. After transferring to a PVDF membrane and blocking by 3% BSA in TBST, the membrane was incubated with anti-TERT antibodies and secondary anti-rabbit HRP conjugated antibodies.

### Statistical analysis

Values are given as the mean ± SE. Data were analyzed by one- and two-way ANOVA, and differences between the control and treated groups were determined using the Bonferroni test. Differences were considered significant at p < 0.05 and determined with GraphPad Prism 7.0 software (La Jolla, CA, USA).
